# Diagnostic Overshadowing of Post-ictal Psychosis in the ED– A Case Series

**DOI:** 10.1192/j.eurpsy.2023.1623

**Published:** 2023-07-19

**Authors:** K. Keenan, A. Moran

**Affiliations:** Department of Psychiatry, Connolly Hospital, Dublin, Ireland

## Abstract

**Introduction:**

Diagnostic overshadowing is established as a mechanism by which physical symptoms are misattributed to mental disorder, hence under-diagnosing and under-treating medical pathology. We report a case series of adult males with established neurological disorders who presented to the ED with visual hallucinations in the postictal period. The phenomena of postictal psychosis is long established in neuropsychiatric literature, with reported rates of postictal psychosis in epilepsy of 2%. Patients vsual hallucinations resolve with anticonvulsant stabilisation and rarely require antipsychotic augmentation.

**Objectives:**

To illustrate diagnostic overshadowing in a case series of postictal psychosis

**Methods:**

Retrospective case series

**Results:**

Case 1:

A 36year old man self-presented to the ED 24hrs post tonic-clonic seizure of 15minutes duration. Medical history was significant for hydrocephalus as an infant with 29 surgical revisions of in-situ ventriculoperitoneal shunt since initial placement. Secondary epilepsy was reported to be poorly controlled with an estimated 50 ED attendances in the past year for management of seizure activity. On assessment new symptomatology of non-threatening visual hallucinations with associated low mood was elicited. A diagnosis of postictal psychosis was advised following psychiatric assessment and medical admission with anticonvulsant titration recommended. Despite this characteristic presentation there were repeated requests to admit this patient to the psychiatric unit and a perceived lack of understanding of his acute medical needs.

Case 2:

A 45year old man self-presented to the ED <24hours post discharge following medical admission for management of seizure. Medical history was significant for a right parieto-temporal infarct one year prior, with acceptable return to functioning following rehabilitation. The man had recently been diagnosed with secondary epilepsy and titration of sodium valproate commenced. The patient presented as distressed in the context of new onset visual hallucinations and palinopsia. Medical admission with urgent neurology input and anticonvulsant titration was advised following psychiatric assessment. ED physician repeatedly stated this patients presentation was stress related and requested psychiatric admission. Following medical admission the patient was managed by neurology. Sodium valproate was augmented with clobazam and the patients psychopathology resolved in full.

**Conclusions:**

Diagnostic overshadowing is prevalent in the ED. Despite established medical diagnoses there may be a reluctance for medical teams to acknowledge or treat organic psychopathology. Psychiatrists must keep abreast of medical comorbidities and physical treatment guidelines of neuropsychiatric disorders in order to advocate appropriately for due medical input. Postictal psychosis is effectively managed by neurological input for effective seizure control with collaborative neuropsychiatry input.

**Disclosure of Interest:**

None Declared

